# Child maltreatment among victims of violent death: an analysis of national violent death reporting system data, 2014–2018

**DOI:** 10.1186/s40621-023-00474-1

**Published:** 2023-11-29

**Authors:** Nicole M. Barrett, Nichole L. Michaels, Sandhya Kistamgari, Gary A. Smith, Farah W. Brink

**Affiliations:** 1 Pediatric Resource Center at Atrium Health Levine Children’s Hospital, 901 East Blvd., Charlotte, NC 28203 USA; 2 Wake Forest University School of Medicine, 475 Vine St., Winston-Salem, NC 27101 USA; 3 The Ohio State University College of Medicine, 370 W 9th Ave, Columbus, OH 43210 USA; 4 The Center for Injury Research and Policy, The Abigail Wexner Research Institute at Nationwide Children’s Hospital, 700 Children’s Drive, Columbus, OH 43215 USA; 5 The Center for Family Safety and Healing at Nationwide Children’s Hospital, 655 E. Livingston Ave., Columbus, OH 43205 USA

**Keywords:** Child maltreatment, Fatalities, Violent death, Suicide, Homicide, Mortality

## Abstract

**Background:**

Limited information is known about the impact of childhood maltreatment on lifetime risk of violent death. This study aimed to compare manner of death, demographics, age at time of death, and the presence of a mental health or substance use disorder among decedents of violent deaths with a history of child maltreatment to those without.

**Methods:**

This cross-sectional study compared characteristics of pediatric and adult violent deaths with and without a history of child maltreatment that were captured in the National Violent Death Reporting System from 2014 through 2018.

**Results:**

Decedents who were male, multiracial, and had adulthood substance or mental health disorders were more likely to have a history of maltreatment. All-age decedents with a history of maltreatment were more likely to die by homicide. Adult decedents with a history of maltreatment were more likely to die by suicide. Maltreated decedents died significantly younger than non-maltreated decedents.

**Conclusions:**

Among victims of violent deaths, an identified history of child maltreatment was associated with increased risk of homicide across the lifespan, adult suicide, and earlier death. A history of child maltreatment was also associated with mental health and substance use disorders, which may reflect one of the pathways through which the child maltreatment-to-death association functions.

## Background

Adverse childhood experiences (ACEs) are known risk factors for poor long-term health across the lifespan and are associated with an increased incidence of adult-onset chronic diseases, substance use, mental health disorders and suicide (Caffo and Belaise 2003; Felitti et al. 1998; Fusco 2021; Grummitt et al. 2021; Kalmakis and Chandler 2015; Kelly-Irving et al. 2013; Petruccelli et al. 2019; Rod et al. 2020; Segal et al. 2021). As one of these early adversities, child maltreatment often occurs within the context of other forms of trauma and psychosocial difficulties (Briggs et al. 2021; Trinidad 2021). Specifically, youth who have been victims of child maltreatment are more likely to have mental health and substance use problems extending into adolescence (Zhu et al. 2023). Childhood victims of maltreatment are known to be at increased risk for fatal child abuse, death related to neglect, and death due to medical causes (Douglas and Lee 2020; Michaels and Letson 2021; Schneiderman et al. 2021; Child Maltreatment 2020). Additionally, children who were involved with child welfare services have higher rates of dying prematurely as they enter adulthood than their peers (Jackisch et al. 2019), although less is known about child maltreatment’s contribution to a lifetime risk of violent death specifically. It is possible that a history of child maltreatment puts individuals at risk for violent or risky behaviors, such as substance use or externalizing harm, which would then predispose them to violent fatalities at an earlier age.

Understanding the link between child maltreatment and suicide, homicide, and unintentional firearm injury deaths would have important prevention and policy implications. Therefore, the objectives of this study were to compare distributions of manner of death, age at time of death, and the presence of a documented mental health or substance use disorder among decedents with a known history of child maltreatment to those without. We hypothesized that decedents of violent death with a history of child maltreatment would be more likely to die by suicide and die at a younger age than decedents of violent death without a known history of child maltreatment.

## Methods

### Data source

Restricted-access data from the five most recent years available at the time of the analysis (2014–2018) in the Center for Disease Control and Prevention’s (CDC) National Violent Death Reporting System (NVDRS) were analyzed. This system gathers death certificate, coroner/medical examiner (C/ME), and law enforcement (LE) reports from violent deaths occurring in the United States (US) and organizes them into an incident-based surveillance system. Trained data abstractors use information from these sources to record more than six hundred standardized variables in the NVDRS web-based system using CDC guidance and definitions. Abstractors also compose two case narratives for each incident, one from the LE report, and one from the C/ME report. These case narratives serve as a summary and provide additional descriptive context of the incident (System and (NVDRS) Web Coding Manual 2016).

The NVDRS began gathering data from six states in 2003, and gradually expanded over time to include all 50 US states, the District of Columbia, and Puerto Rico. Eighteen states contributed data in 2014, with this number increasing to forty-one states by 2018. Because more states participated in the NVDRS in later years, we decided to limit our data sample to the five most recent years available at the time we requested the data. Data for this study come from the following states: Alaska, Colorado, Georgia, Kentucky, Maryland, Massachusetts, Michigan, New Jersey, New Mexico, North Carolina, Ohio, Oklahoma, Oregon, Rhode Island, South Carolina, Utah, Virginia, and Wisconsin (2014–2018); Hawaii (2015–2016); Arizona, Connecticut, Kansas, Maine, Minnesota, New York, and Vermont (2015–2018); Illinois, Indiana, Iowa, Pennsylvania, and Washington (2016–2018); California, Delaware, District of Columbia, Nevada, New Hampshire, Puerto Rico, and West Virginia (2017–2018); Alabama, Louisiana, Missouri, and Nebraska (2018). The 2017 data for California includes violent deaths that occurred in four of its fifty-eight counties (Los Angeles, Sacramento, Shasta, and Siskiyou). The 2018 data for California includes violent deaths that occurred in twenty-one counties. All other states provided data from all areas (System and (NVDRS) Web Coding Manual 2016).

The NVDRS is a unique data source for information about violent deaths. By utilizing data from multiple types of investigative reports, the NVDRS is able to provide detailed information about violent deaths not captured elsewhere. The inclusion of case narratives based on information provided in LE and C/ME reports provides additional detail beyond what is coded in NVDRS variables.

### Case selection

NVDRS data for all violent deaths occurring from January 1, 2014 through December 31, 2018 were obtained from the CDC. Cases were classified into two groups: those who had an identified history of child maltreatment and those who did not. A history of child maltreatment was determined using two methods. The first was using the NVDRS abstractor-coded variable “history of abuse or neglect as a child,” which was coded as “yes” if the victim had a history of abuse (physical, sexual, or psychological), neglect (physical, medical/dental, emotional, or educational), or exposure to violent environments or inadequate supervision as a child (Jackisch et al. 2019). The abstractors were instructed to use the “history of abuse or neglect as a child” variable only if there was no direct link to the violent death or the link was unknown. They were specifically instructed not to code this variable if the abuse or neglect directly caused or precipitated the death. In order to capture any history of child maltreatment among victims that may not have been identified using this variable, we also manually reviewed case narratives from C/ME and LE reports provided in the NVDRS. One author XX used key phrases to search the case narratives, including child[hood] abuse/maltreatment, physical abuse, sexual abuse/assault, molest, rape, neglect, psychological abuse/maltreatment, CPS, children services, and child protective services. If one or more of the key phrases were identified in a given case narrative, XX used the CDC definition of child maltreatment to determine if the case met criteria for childhood maltreatment. According to the CDC, child maltreatment is defined as “any act or series of acts of commission or omission by a parent or other caregiver that results in harm, potential for harm, or threat of harm to a child.” The CDC further defines a caregiver as:

“a person, or people, who at the time of the maltreatment is in a permanent (primary caregiver) or temporary (substitute caregiver) custodial role. In a custodial role, the person is responsible for care and control of the child and for the child’s overall health and welfare [and] can include clergy, coaches, teachers, relatives, babysitters, residential facility staff, or others who are not the child’s primary caregiver(s) (Leeb et al. 2008).”

All case narratives that indicated the child maltreatment was related to the case fatality were excluded. A random selection of 3,890 (2%) of cases were reviewed and coded independently by an additional author YY. Interrater reliability was assessed using a Cohen’s kappa statistic, which indicated near perfect agreement (kappa = 0.93). All discrepancies identified in the paired coding process were discussed and recoded based on mutual consensus, with additional input from author ZZ as needed.

The following NVDRS-coded variables were included in the analyses: victim’s manner of death, age, sex, race, ethnicity, homelessness, and education level. Additional variables indicating the presence or absence of a mental health disorder or substance use disorder were created, based on available NVDRS-coded variables. For this study, the victim was considered to have a mental health disorder if one or more of the following variables were affirmed: currently having a mental health problem, currently in treatment for a mental health problem, had a history of ever being treated for a mental health problem, or if they were currently in a crisis due to a diagnosed mental health problem. The victim was considered to have a substance use disorder if one or more of the following NVDRS abstractor-coded variables were indicated: they were identified as having an alcohol dependence or other substance abuse problem, or if they were currently in a crisis due to an alcohol dependence or other substance use problem.

All analyses were conducted using SAS 9.4 (SAS Institute, Inc., Cary, NC). Categorical variables were described in terms of frequency and proportion while continuous variables were described in terms of mean and standard deviation. Analyses for this study focused primarily on comparing cases with an identified history of child maltreatment to those without such history. Comparisons between these groups were made using Chi square tests of association. Post-hoc chi-square tests were used when multiple comparisons were needed. To assess the magnitude of these associations, univariate logistic regression models were employed using history of child maltreatment as dependent variable and all the other categorical variables as independent variables. Univariate and multivariate logistic regression models were used to investigate the relationship between history of child maltreatment and the manner of death. In these models, history of child maltreatment was treated as the independent variable, while the manner of death was treated as the dependent variable with sex, race, ethnicity, education level and homelessness added as covariates of interest.

Furthermore, the relationship between age at the time of death and history of maltreatment was analyzed using univariate and multivariate linear regression models, considering age at death as the dependent variable and history of maltreatment as the independent variable and other variables such as sex, race, ethnicity, education level and homelessness were included as other covariates. However, education level and homelessness were not included as covariates for any analysis among victims < 18 years as they were not meaningful in this age group. Likewise, mental health disorders and substance use disorders were omitted from the regression models as they are part of the suggested causal pathway. Statistical level of significance was set at a *p* value of ≤ 0.05 for all analyses. This study was considered exempt by the institutional review board at the authors’ institution.

## Results

**Fig. 1:**
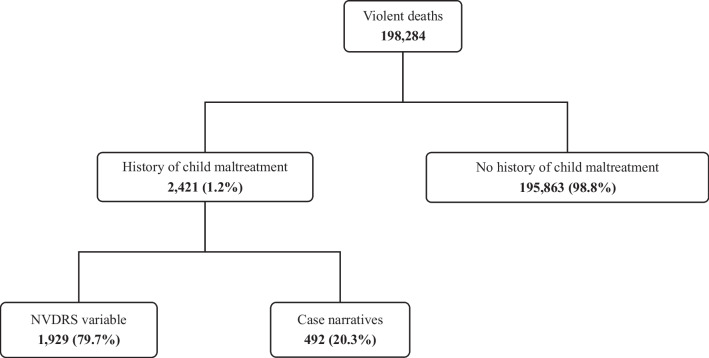
Case selection of violent death decedents in the NVDRS, 2014–2018

There were 198,284 violent deaths reported to the NVDRS during the study timeframe and 2,421 (1.2%) decedents had a known history of child maltreatment. Of this total, 1,929 (79.7%) cases were identified using the NVDRS variable “history of abuse or neglect as a child” and an additional 492 (20.3%) cases were identified by review of the narratives from C/ME and LE reports (Fig. 1). The mean age at time of death for the maltreatment group was 24.8 years (SD 19.6, range 6–112). Most decedents were white (75.7%) and not Hispanic (89.8%). While more than three-quarters (77.3%) of decedents in the non-maltreated group were male, fatalities were more evenly distributed between male (54.6%) and female (45.4%) decedents with a known history of child maltreatment. Among adult decedents with history of child maltreatment, the majority had a documented mental health disorder (79.2%) and approximately one-half had a substance use disorder (48.9%; Table 1).

**Table 1:** Descriptive Characteristics of Decedents of Violent Death with and without a History of Child Maltreatment, NVDRS 2014–2018, *N *= 198,284

Decedent characteristics	History of maltreatment, *n* (%)	No history of maltreatment, *n* (%)	Chi-square	*p* value
Age (in years)				
< 18 years	1005 (41.5)	8904 (4.6)	6883.04	< 0.0001
≥ 18 years	1416 (58.5)	186,959 (95.5)		
Manner of death				
Suicide	1486 (61.4)	124,497 (63.6)	34.68	< 0.0001
Homicide	701 (29.0)	48,514 (24.8)		
Unintentional firearm	15 (0.6)	1234 (0.6)		
Legal intervention	12 (0.5)	2616 (1.3)		
Undetermined	207 (8.6)	19,002 (9.7)		
Sex				
Male	1323 (54.6)	151,410 (77.3)	694.45	< 0.0001
Female	1098 (45.4)	44,439 (22.7)		
Unknown	0	14		
Race				
White	1830 (75.7)	144,650 (74.0)	102.49	< 0.0001
Black or African American	394 (16.3)	38,849 (19.9)		
American Indian/Alaska Native	48 (2.0)	2941 (1.5)		
Asian/Pacific Islander	45 (1.9)	4354 (2.2)		
Other/Unspecified	29 (1.2)	2719 (1.4)		
Two or more	70 (2.9)	1997 (1.0)		
Unknown	5	353		
Ethnicity				
Hispanic	242 (10.2)	176,759 (91.1)	6.49	0.039
Not Hispanic	2127 (89.8)	17,210 (8.9)		
Unknown	0	1894		
Mental health disorder, age ≥ 18 years				
Yes	1121 (79.2)	65,603 (35.1)	1193.61	< 0.0001
No	295 (20.8)	121,356 (64.9)		
Substance use disorder, age ≥ 18 years				
Yes	693 (48.9)	51,178(27.4)	327.59	< 0.0001
No	723 (51.1)	135,781(72.6)		

Decedents of violent deaths who were male (OR 2.83, 95% CI 2.61–3.07), multiracial (OR 2.77, 95% CI 2.17–3.53), or who had a substance use disorder (OR 2.35, 95% CI 2.17–2.54) or a mental health disorder (OR 1.23, 95% CI 1.13–1.34) were more likely to have a documented history of child maltreatment than decedents those who were female, white, or who did not have a substance use disorder or mental health disorder. (Table 2). Decedents who were Black/African American (OR 0.80, 95% CI 0.72–0.89) or not Hispanic (OR 0.84, 95% CI 0.74–0.96) were less likely to have an identified history of child maltreatment than decedents who were white and Hispanic, respectively. Among decedents who were < 18 years old at time of death, those who were Asian/Pacific Islander were less likely to have a known history of child maltreatment than those who were white (OR 0.47, 95% CI 0.27–0.81), however among decedents who were ≥ 18 years old at time of death, those who were Black/African American were less likely to have a history of child maltreatment (OR 0.33, 95% CI 0.27–0.40) than those who were white. All-age decedents with an education level of 8th grade or less were more likely to have a known history of child maltreatment than those with a doctorate or professional degree (OR 11.06, 95% CI 7.00–17.46), but among decedents ≥ 18 years old at time of death, decedents with an education level of 8^th^ grade or less were less likely to have a known history of maltreatment than those with a doctorate or professional degree (OR 0.52, 95% CI 0.28–0.95; Table 2).

**Table 2:** Comparison of Decedents of Violent Death with a History of Child Maltreatment versus Without, NVDRS 2014–2018

	All ages	< 18 years old	≥ 18 years old
Decedent characteristics	OR (95% CI)	OR (95% CI)	OR (95% CI)
Sex			
Female	REF	REF	REF
Male	2.83 (2.61–3.07)	2.05 (1.80–2.34)	2.85 (2.57–3.17)
Age (in years)			
< 18 years old	REF	–	–
≥ 18 years old	0.07 (0.06–0.07)		
Race			
White	REF	REF	REF
Black or African American	0.80 (0.72–0.89)	0.92 (0.79–1.06)	0.33 (0.27–0.40)
American Indian/Alaska Native	1.29 (0.97–1.72)	1.12 (0.74–1.70)	0.92 (0.6 -1.41)
Asian/Pacific Islander	0.82 (0.61–1.10)	0.47 (0.27–0.81)	0.87 (0.61–1.24)
Other/Unspecified	0.84 (0.58–1.22)	0.52 (0.30–0.92)	0.73 (0.45–1.20)
Two or more	2.77 (2.17–3.53)	1.57 (1.13–2.17)	1.58 (1.05–2.37)
Ethnicity			
Hispanic	REF	REF	REF
Not Hispanic	0.84 (0.74–0.96)	1.08 (0.89–1.31)	1.08(0.89–1.31)
Education^a^			
Doctorate or professional degree	REF	–	REF
8th grade or less	11.06 (7.00–17.46)	–	0.52 (0.28–0.95)
9th to 12th grade, no diploma	1.59 (1.00–2.53)	–	0.84 (0.52–1.36)
High school graduate/GED	0.96 (0.61–1.52)	–	0.94 (0.60–1.49)
Some college credit, no degree	1.17 (0.73–1.87)	–	1.17 (0.73–1.87)
Associate’s degree	1.30 (0.80–2.11)	–	1.30 (0.80–2.11)
Bachelor’s degree	1.12 (0.69–1.80)	–	1.12 (0.69–1.80)
Master’s degree	1.18 (0.70–2.00)	–	1.18 (0.70–2.00)
Presence of mental health disorder	2.35 (2.17–2.54)	0.94 (0.80–1.10)	7.03 (6.18–7.99)
Presence of substance use disorder	1.23 (1.13–1.34)	0.90 (0.66–1.22)	2.54 (2.29–2.82)

^a^Education level was not applicable to majority of victims < 18 years old

After adjusting for the sex, race and ethnicity, decedents of all ages with an identified history of child maltreatment were less likely to die from suicide (AOR 0.89, 95% CI 0.81–0.98) and legal intervention deaths (AOR 0.38, 95% CI 0.20–0.74) and more likely to die by homicide (AOR 1.41, 95% CI 1.27–1.58) compared with those without a history of child maltreatment. Decedents ≥ 18 years old at time of death with an identified history of child maltreatment were more likely to die from suicide (AOR 3.36, 95% CI 2.82–4.02) and less likely to die by homicide (AOR 0.24, 95% CI 0.18–0.30) and legal intervention (AOR: 0.43, 95%CI: 0.22–0.82) compared with those without a history of child maltreatment, after adjusting for sex, race, ethnicity, education level, and homelessness. After adjusting for sex, race and ethnicity, decedents < 18 years old at time of death with a known history of maltreatment were less likely to die from suicide (AOR 0.33, 95% CI 0.28–0.38) and unintentional firearm deaths (AOR 0.31, 95% CI 0.17–0.56; Table 3) and more likely to die from homicide (AOR 2.85, 95% CI: 2.46–3.31) than those without a history of child maltreatment.

**Table 3:** Comparison of manners of violent death of decedents with a history of child maltreatment versus without, NVDRS 2014–2018

	Unadjusted—Coefficient	Unadjusted OR (95%CI)	Adjusted-Coefficient	Adjusted OR (95%CI)
Suicide, all ages	− 0.094	0.91 (0.84–0.99)	− 0.109	0.89 (0.81–0.98)^a^
Victim age ≥ 18 years	1.234	3.44 (2.95–3.99)	1.213	3.36 (2.82–4.02)^b^
Victim age < 18 years	− 0.941	0.39 (0.34–0.45)	− 1.125	0.33 (0.28–0.38)^a^
Homicide, all ages	0.213	1.24 (1.13–1.35)	0.347	1.41 (1.27–1.58)^a^
Victim age ≥ 18 years	− 1.614	0.20 (0.16–0.25)	− 1.447	0.24 (0.18–0.30)^b^
Victim age < 18 years	0.876	2.40 (2.10–2.75)	1.048	2.85 (2.46–3.31)^a^
Unintentional firearm, all ages	− 0.017	0.98 (0.59–1.64)	0.074	1.08 (0.63–1.83)^a^
Victim age ≥ 18 years	− 0.783	0.46 (0.15–1.42)	− 0.565	0.57 (0.18–1.77)^b^
Victim age < 18 years	− 1.275	0.28 (0.16–0.50)	− 1.170	0.31 (0.17–0.56)^a^
Legal intervention, all agesc	− 0.998	0.37 (0.21–0.65)	− 0.959	0.38 (0.20–0.74)^a^
Victim age ≥ 18 years	− 0.489	0.61 (0.35–1.08)	− 0.857	0.43 (0.22–0.82)^b^

^a^Controlling for sex, race and ethnicity

^b^Controlling for sex, race, ethnicity, education level, and homelessness

^c^The ORs for legal intervention deaths < 18 years were unstable

**Fig. 2:**
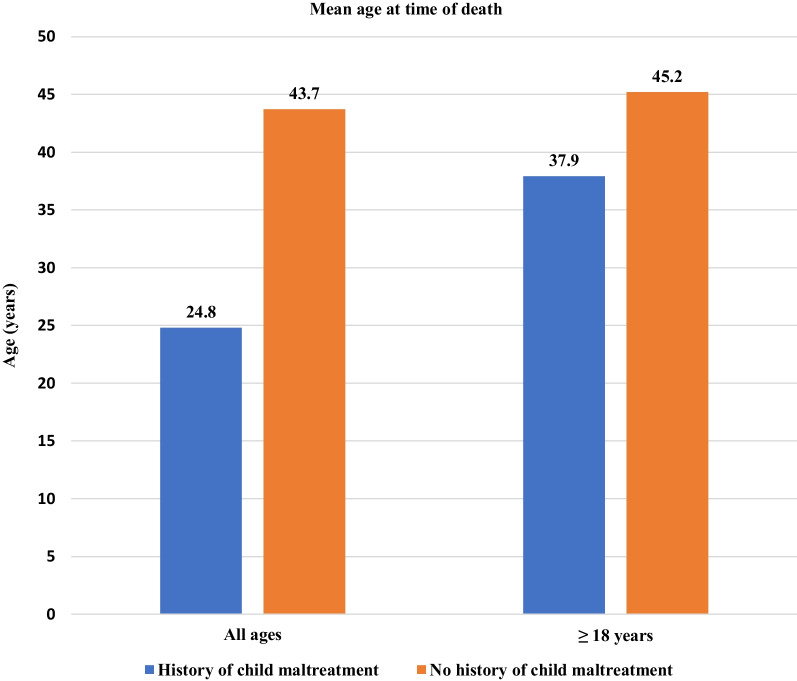
Comparison of mean age at time of violent death between maltreatment and non-maltreatment groups

Decedents in the NVDRS with a known history of child maltreatment died significantly younger (mean = 24.8 years) than non-maltreated decedents (mean = 43.7 years; *p* < *0.001*; Fig. 2). When limiting this analysis to decedents ≥ 18 years old at time of death, this relationship remained statistically significant (*p* < *0.001*) with the maltreated group dying at a mean age of 37.9 years (vs. 45.2 years in the non-maltreated group; Fig. 2). After adjusting for sex, race, ethnicity, education level and homelessness for the adult cohort, the mean difference in age at the time of death was 18.5 years for all ages (*p* < *0.001*), 8.2 years for those ≥ 18 years old (*p* < *0.001*), and 5.5 years for those < 18 years old (*p* < *0.001;* Table 4).

**Table 4:** Difference in Mean Age at Time of Violent Death of Decedents with a History of Child Maltreatment vs. Without, NVDRS 2014–2018

	Unadjusted		Adjusted	
	Mean difference	*p* value	Mean difference	*p* value
All ages	− 18.9	< 0.001	− 18.5	< 0.001^a^
Victim age ≥ 18 years	− 7.2	< 0.001	− 8.2	< 0.001^b^
Victim age < 18 years	− 5.9	< 0.001	− 5.5	< 0.001^a^

^a^Controlling for sex, race and ethnicity

^b^Controlling for sex, race, ethnicity, education level, and homelessness

## Discussion

Among our study population, 1.2% of victims of violent death had an identified history of child maltreatment, and like individuals without a history of maltreatment, most died by suicide. Overall, decedents of violent death who were male or multiracial were more likely to have a documented history of child maltreatment. Further, decedents with a history of child maltreatment died at significantly younger ages than those without an identified history of maltreatment.

### Child maltreatment and substance use or mental health disorders

In our study, all-age and adult decedents of violent deaths who had a reported substance use disorder were more likely to have an identified history of child maltreatment. This is consistent with prior research, which has shown that individuals who were exposed to ACEs, and child maltreatment specifically, have increased risk of substance use problems (Felitti et al. 1998; Fusco 2021; Kalmakis and Chandler 2015; Leeb et al. 2008; Afifi et al. 2012; Banducci et al. 2014; Fenton et al. 2013; Kisely et al. 2021). In addition, decedents who had a mental health disorder as an adult were more likely to have a history of child maltreatment. This is also consistent with prior research demonstrating that maltreated children are at increased risk for several behavioral and emotional difficulties, including Post-Traumatic Stress Disorder, mood disorders such as anxiety and depression, disruptive behavioral disorders, and high-risk behaviors including self-harm and suicidal behaviors (Kalmakis and Chandler 2015; Petruccelli et al. 2019; Segal et al. 2021; Bellis 2002; Fry et al. 2012; Lindert et al. 2014; Perepletchikova and Kaufman 2010; Ruch et al. 2021; Turner and Colburn 2022). The relationship between early adversity and future substance use or mental health disorders is thought to stem, in part, from the development of risky behaviors and secondary trauma resulting from the victim’s early traumatic experiences (Day et al. 2013). While the relationship between child maltreatment and substance use or mental health disorders is not a novel finding, this study adds to the growing body of literature supporting long-term treatment programs for this unique population (Zhu et al. 2023).

### Child maltreatment and homicide

In the current study, all-age decedents of violent deaths with an identified history of child maltreatment were more likely to die by homicide than those without a known history of child maltreatment. Prior studies have reported on child homicides that are the direct result of abuse or neglect or that are related to intimate partner violence (Douglas and Lee 2020; Michaels and Letson 2021; Adhia et al. 2019; Hunter et al. 2021; Jonson-Reid et al. 2007; Putnam-Hornstein 2011; Sabotta and Davis 1992; Sorenson and Peterson 1994). The current study specifically evaluated violent deaths that were not the direct result of abuse or neglect during childhood. To our knowledge, this is the first study to characterize adult victims of child maltreatment who became homicide victims as adults or during homicide incidents that were not a direct result of child abuse or neglect. This is likely due to the multifactorial risk factors for child maltreatment which also influence a person’s risk of becoming a victim of violence into adulthood, including low socioeconomic status, poor social support, and living in communities with high levels of crime (Garbarino and Sherman 1980; Kim and Drake 2023). Societal prevention efforts targeted to these risk factors, such as supplemental income benefits, may have a greater effect in reducing the incidence of homicide in the maltreated population.

### Child maltreatment and suicide

Our study identified that decedents of violent deaths ≥ 18 years old at time of death with an identified history of child maltreatment were more likely to die by suicide compared with those without an identified history of child maltreatment. In the US, suicide ranks as the second leading cause of death for individuals 10–34 years old and the tenth leading cause of death among all age groups (National Center for Injury Prevention and Control C 2018). A recent systematic review found that childhood adversity was associated with 38% of adult suicide deaths in the US (Grummitt et al. 2021). While there is a relative paucity of research on causal risk factors for suicide, prospective studies and twin studies have demonstrated a relationship between maltreatment during childhood and suicide (Brown et al. 1999; Cha et al. 2018; Fergusson et al. 1996, 2008; Liu et al. 2017; Nelson et al. 2002). Prior research has evaluated the relationship between child abuse and suicidality in terms of suicidal thoughts, attempts, and self-injury (Liu et al. 2017; Angelakis et al. 2019; Castellví et al. 2017; Johnson et al. 2002; Klonsky and Moyer 2008; Martin et al. 2016; Miller et al. 2013; Tatnell et al. 2017; Wong et al. 2020; Zatti et al. 2017).

Few studies have examined the relationship between child maltreatment and actual deaths by suicide and none were conducted within the past decade. In a small cohort, Shafii et al. (Shafii et al. 1985) compared twenty children and adolescents who died by suicide to a matched-pair control group and found that those who died by suicide were more likely to have had parental absence or physical or emotional abusiveness. A larger study utilizing psychological autopsy data found that a lifetime history of abuse was a significant risk factor for suicide, although this finding was limited by these data only being available in a subset of the suicides evaluated (Brent et al. 1999). Many studies examining suicide and history of child maltreatment are limited to a youth or early adult sample (Brown et al. 1999; Cha et al. 2018; Fergusson et al. 1996, 2008; Singh and Lathrop 2008). The current study updates this prior work with recent findings, as well as contributes to our knowledge regarding suicide across the lifespan. Long-term mental health support among child maltreatment victims may be an effective suicide prevention strategy among this population (Impact of mental health treatment on suicide attempts,McClellan 2021).

### Child maltreatment and mortality

A significant finding of this study was that a known history of child maltreatment was associated with violent deaths at an earlier age. In the adjusted regression model, the mean difference in age at time of death was 18.5 years for decedents of violent death of all ages and 8.2 years for those ≥ 18 years old. This finding builds on prior studies, which report that individuals with child protective services contact have been shown to have higher death rates and higher rates of dying earlier than those without child protective services contact (Segal et al. 2021; Jackisch et al. 2019). These studies are limited by using child protective services involvement as an approximation of maltreatment, which is an underestimation of its true prevalence, because not all cases rise to the level of child protective services involvement or substantiation. By using data elements contained in LE and C/ME reports as well as case narratives, the current study captures a wider group of individuals, to whom child maltreatment was thought to have occurred, irrespective of formal child protective services involvement or substantiation. Chen et al. (Chen et al. 2016) found that females, who were self-reported victims of childhood emotional or physical abuse, had increased all-cause mortality. The findings of the current study add that child maltreatment is associated with an individual’s lifetime risk of early violent death specifically. Programs and policies that reduce and prevent violent deaths, such as suicide prevention efforts (Resources 2021) may have an even greater effect on the maltreatment population. Focusing these efforts on children and adults who have a history of child maltreatment may have a significant impact on prevention of violent deaths.

### Study limitations

There are several limitations in our study. First, all 50 states did not contribute data to the NVDRS during this study period. By 2018, 41 states were reporting to the NVDRS, but only 18 reported during the entire time period captured in this study. Thus, our findings may not be generalizable to the entire US population that experienced a violent death. Further, our findings are only applicable to the population who have suffered a violent death and not the overall population.

Second, the NVDRS relies on data provided by C/ME and law enforcement reports, which may not always report the presence or absence of maltreatment accurately, particularly among adult decedents, for whom maltreatment may have occurred in the distant past. Further, certain important factors were unable to be fully evaluated due to the limited information available. For example, we were unable to access data on certain socioeconomic factors, concurrent adverse life events, law enforcement involvement, and historical abuse details. We controlled for the effect of homelessness and education level, but were unable to control for other factors, such as social support or community crime levels, which are known risk factors for both child maltreatment and violence. Specifically, the NVDRS contained limited details on the type and perpetrator of historical abuse, whether the decedent was a victim of one or more types of maltreatment, as well as details regarding the severity and chronicity of the abuse, and when the maltreatment occurred. History of mental health and substance use disorders documented in the NVDRS may be based on information from individuals who knew the victim or circumstances identified in the death investigation, rather than official medical records. The case narratives within the NVDRS may also be a source of information bias, as certain decedent characteristics (e.g., age, race/ethnicity) may be more likely to have missing text across LE and C/ME narratives. While we are not aware of a consistent association with missing text across specific demographics, the authors acknowledge the possibility of missing, incomplete, or incorrect narrative data (Mezuk et al. 2021).

Finally, although we identified additional cases of child maltreatment by utilizing case narratives, the true prevalence is likely higher. This study is also limited by the lack of a comparison group (i.e., individuals with or without a history of child maltreatment who did not die a violent death).

## Conclusions

This study identified that victims of violent deaths with a history of child maltreatment were more likely to die by homicide and suicide as adults, as well as were more likely to die earlier and have mental health or substance use disorders. Additional studies using a nationally representative sample with potential confounding variables are needed to confirm these results.

### Disclaimer

This research uses data from NVDRS, a surveillance system designed by the Centers for Disease Control and Prevention's (CDC) National Center for Injury Prevention and Control. The findings are based, in part, on the contributions of the funded states and territories that collected violent death data and the contributions of the states' partners, including personnel from law enforcement, vital records, medical examiners/coroners, and crime laboratories. The analyses, results, and conclusions presented here represent those of the authors and do not necessarily reflect those of CDC. Persons interested in obtaining data files from NVDRS should contact CDC's National Center for Injury Prevention and Control, 4770 Buford Hwy, NE, MS F-64, Atlanta, GA 30341–3717, (800) CDC-INFO (232–4636; CDC, 2016).

## Data Availability

The data that support the findings of this study are available from the Centers for Disease Control and Prevention (CDC), but restrictions apply to the availability of these data, which were used under license for the current study, and therefore are not publicly available. However, data are available from the authors upon reasonable request and with permission from the Centers for Disease Control and Prevention.

## Abbreviations

ACEs: Adverse childhood experiences
CDC: Centers for disease control and prevention
CI: Confidence interval
C/ME: Coroner/medical examiner
LE: Law enforcement
NVDRS: National Violent Death Reporting System
OR: Odds ratio
